# Internet and mobile technologies: addressing the mental health of trauma survivors in less resourced communities

**DOI:** 10.1017/gmh.2017.11

**Published:** 2017-08-30

**Authors:** J. I. Ruzek, C. M. Yeager

**Affiliations:** 1 National Center for PTSD Dissemination and Training Division, Palo Alto, California, USA; 2 VA Palo Alto Health Care System, Palo Alto, California, USA; 3 M^2^ Institute at Palo Alto University, Palo Alto, California, USA; 4 Department of Psychiatry and Behavioral Sciences, Stanford University, Palo Alto, California, USA; 5 Psychology Department, University Colorado Colorado Springs, Colorado Springs, Colorado, USA; 6 Trauma, Health, & Hazards Center, University of Colorado Colorado Springs, Colorado Springs, Colorado, USA

**Keywords:** Low- and middle-income countries (LMIC), mental health, mobile health (mHealth), post-traumatic stress disorder (PTSD), technology, trauma

## Abstract

Internet and mobile technologies offer potentially critical ways of delivering mental health support in low-resource settings. Much evidence indicates an enormous negative impact of mental health problems in low- and middle-income countries (LMICs), and many of these problems are caused, or worsened, by exposure to wars, conflicts, natural and human-caused disasters, and other traumatic events. Though specific mental health treatments have been found to be efficacious and cost-effective for low-resource settings, most individuals living in these areas do not have access to them. Low-intensity task-sharing interventions will help, but there is a limit to the scalability and sustainability of human resources in these settings. To address the needs of trauma survivors, it will be important to develop and implement Internet and mobile technology resources to help reduce the scarcity, inequity, and inefficiency of current mental health services in LMICs. Mobile and Internet resources are experiencing a rapid growth in LMICs and can help address time, stigma, and cost barriers and connect those who have been socially isolated by traumatic events. This review discusses current research in technological interventions in low-resource settings and outlines key issues and future challenges and opportunities. Though formidable challenges exist for large-scale deployment of mobile and Internet mental health technologies, work to date indicates that these technologies are indeed feasible to develop, evaluate, and deliver to those in need of mental health services, and that they can be effective.

Much evidence indicates an enormous negative impact of mental health problems in low- and middle-income countries (LMICs; World Health Organization, 2001). Mental health disorders are the leading cause of global disability burden (Whiteford *et al.* 2015), accounting for about 14% of the global burden of disease (World Health Organization, 2010). Three-quarters of the disease burden that is due to mental, neurological, and substance use disorders affects LMICs (Lopez *et al.* 2006). Mental disorders also have an impact on mortality, accounting for 1–2 million deaths every year (Prince *et al.* 2007).

Many of these problems – post-traumatic stress reactions, depression, anxiety, substance abuse – are caused, or worsened, by exposure to wars, conflicts, natural and human-caused disasters, and other traumatic events. Millions of people around the world continue to encounter these stressors, sometimes on an ongoing basis, and this exposure may be expected to increase in coming years due to the continuing effects of mass migration and climate change (USGCRP, 2016). In 2013, it was estimated that 148.2 million people were affected by natural disasters or displaced by conflict (United Nations Office for the Coordination of Humanitarian Affairs, 2014). In a World Health Organization (WHO) study of 21 countries (Stein *et al.* 2010), more than 10% of respondents reported witnessing violence (21.8%) or experiencing interpersonal violence (18.8%), accidents (17.7%), exposure to war (16.2%), or trauma to a loved one (12.5%). Torture is endemic in countries affected by pervasive conflict, with 21% of participants in 84 surveys reporting personal experiences of torture (Steel *et al.* 2009). Gray *et al.* (2016) highlighted the prevalence of potentially traumatic experiences (PTEs) in their study of orphaned and separated adolescents in five LMICs, finding that nearly all (90%) of participants reported experiencing at least one lifetime PTE. In a comprehensive review of survivors of mass conflict and displacement, Steel *et al.* (2009) confirmed a dose–response effect for exposure to potentially traumatic events in relation to both depression and post-traumatic stress disorder (PTSD). Their meta-analysis of post-conflict studies using representative samples and full diagnostic assessment found that 15.4% of people reported PTSD and 17.3% reported depression (Steel *et al.* 2009).

Costs associated with these conditions are widespread and include economic loss, stigma and discrimination, and massive disruption of the quality of life of both the individual and their families (WHO, 2001). Often, trauma is not only an individual experience, but also a collective one. In natural disasters and wars, entire communities are often traumatized. Modern forms of terrorism not only seek to destroy life but ways of life, and can sometimes abolish the sense of community that is important to recovery (Somasundaram, 2010). Awareness of the mental health impact of trauma has led to the establishment of the WHO Guidelines for the Management of Conditions Specifically Related to Stress, which outlines recommendations for the assessment and management of acute stress, PTSD, and grief and prolonged grief disorder (Tol *et al.* 2013).

Though specific mental health treatments have been found to be efficacious and cost-effective for low-resource settings (Barry *et al.* 2013), most individuals living in these areas do not have access to them (Kohn *et al.* 2004; Saxena *et al.* 2007). The WHO World Mental Health Survey Consortium (2004) reported that 76–85% of those with serious mental health disorders received no treatment in LMICs. Only a very small proportion of the health budget in LMICs is allocated to the treatment and prevention of these disorders, averaging 1.9% in lower middle-income countries and 0.5% in low-income countries) (Jacob *et al.* 2007). As a result, barriers to psychological treatments in these settings include a lack of skilled human resources, limited funding and infrastructure, centralization and inequitable distribution of services, social stigma associated with help-seeking, and cultural appropriateness and acceptability (Kohn *et al.* 2004; Saraceno *et al.* 2007; Patel *et al.* 2011). Kakuma *et al.* (2011) found a serious shortfall of human resources for mental health in LMICs that is likely to grow unless effective steps are taken. Looking across 58 LMICs, Bruckner *et al.* (2010) estimated that an adequate mental health response capacity would require 20 000 psychiatrists, 195 000 nurses, and 147 000 psychosocial care providers.

In this paper, we explore evolving strategies for strengthening mental health response to the needs of trauma survivors in LMICs and other low-resource settings. We describe some emerging trauma-related technology interventions, and identify some key challenges related to their cultural adaptation, getting them in the hands of trauma survivors, using them to help build the sense of community and capacity for social advocacy that underpin mental health, and developing the methods and systems for implementation of technology-facilitated mental health response. Finally, we present a call to action for mental health policymakers, practitioners, and researchers: to speed the use of emerging Internet and mobile technologies to aid those efforts.

## Mental health response strategies in low resource settings

To improve responses for trauma survivors in the face of such major human resource limitations, it will be necessary for less specialized community workers and family members to help deliver mental health services (Padmanathan & Silva, 2013). This ‘task-sharing’ approach is well underway and represents a paradigm shift (Collins *et al.* 2011; Rosen *et al.* 2016). Research suggests that laypersons can deliver evidence-based treatments (EBTs) for PTSD, if trained and supervised. For example, Bass *et al*. (2013) trained lay women to use Cognitive Processing Therapy (CPT) to treat PTSD, depression, and anxiety symptoms in female survivors of sexual violence in eastern Democratic Republic of Congo. In this study, CPT was found to be effective, despite illiteracy of many of those treated and ongoing exposure to conflict.

Although interventions like CPT, developed and tested in high-resource environments, are likely to be effective, they require intensive training and significant time to deliver (e.g. Beidas & Kendall, 2010; Rosen *et al.* 2017). Critical to making effective mental health services available globally is the development of low-intensity psychological interventions. To be scalable, mental health interventions will need to be capable of delivery by paraprofessionals or peers, relatively brief, focused as much as possible on self-management, and able to address multiple problems at the same time (Bockting *et al.* 2016). The trans-problem/trans-diagnostic criterion is especially important because the various mental health problems are highly co-morbid and it will not be feasible to train paraprofessionals in multiple protocols or field large groups of paraprofessional helpers that each specialize in a different problem (Murray & Jordans, 2016).

To support task-sharing while providing access to quality care, the WHO has begun to develop and test low-intensity cross-diagnostic psychological interventions in low-resource settings (WHO, 2010). A range of low-intensity psychological interventions targeting a number of mental health disorders experienced by trauma survivors is under development. Early trials suggest that they will be effective when delivered by paraprofessionals (Bolton *et al.* 2014*a*, *b*; Patel & Saxena, 2014; Weiss *et al.* 2015; Rahman *et al.* 2016). For example, Problem Management Plus (PM+; Dawson *et al.* 2015) was recently found to be effective in improving functioning and decreasing PTSD symptoms in a conflict-affected area of Pakistan (Rahman *et al.* 2016). Similarly, the Common Elements Treatment Approach (CETA; Murray *et al.* 2013) has shown promising results for treatment of symptoms of anxiety, depression, and PTSD in Burmese refugees (Bolton *et al.* 2014*a*, *b*) and survivors of systematic violence in Southern Iraq (Weiss *et al.* 2015).

## Technologies for trauma survivors

An extended workforce represents one critical component in addressing mental health in low-resource environments. However, there is a limit to the scalability and sustainability of human resources in these settings. A meta-analytic review of the acceptability and feasibility of task-sharing concluded that task-sharing alone is not an outright solution for the mental health treatment gap and cited worker distress, competence, incentives, and acceptance as barriers (Padmanathan & Silva, 2013). Training and supervision structures may be critical to effectiveness, which poses an additional burden on human resources.

To address these shortcomings, it may be necessary to go beyond the mobilization of a wide range of human resources to include the use of additional, ‘non-consumable’ technology resources to help reduce the scarcity, inequity, and inefficiency of current mental health services in LMICs. The relative simplicity and brevity of emerging trans-diagnostic interventions suggests that it will be feasible to adapt them for delivery via technology. Technology can help address time, stigma, and cost barriers (Patel, 2014) and connect those who have been socially isolated by traumatic events.

### Technology access in LMICs

At the current time, about 40% of the global population has access to the Internet, there are almost as many mobile phone subscriptions as there are human beings, the rate of mobile cellular subscriptions is 89 per 100 inhabitants in developing nations (compared with 128 in developed nations), and smartphone ownership is rapidly increasing (International Telecommunications Union, 2014). Ownership of smartphones in emerging and developing nations climbed from a median of 21% in 2013 to 37% in 2015, with overwhelming majorities in almost every nation owning some form of mobile device (Pew Research Center, 2016).

### Technologies for mental health

As mobile technologies are becoming used for a comprehensive range of daily tasks (e.g. shopping, banking, and socializing), there is limited but growing evidence for the efficacy of mobile health-related interventions in LMICs (Hall *et al.* 2014). Integration of technology into mental health services is also taking place, via telephone services, video teleconferencing, Internet-based interventions, text messaging, and mobile phone- and smartphone-based interventions. Users keep their phones at hand and turned on, providing opportunities for ‘just-in-time’ support (e.g. managing immediate distress and providing emergency information). Mobile phones, especially, can extend the geographic reach of mental health services in LMICs and provide potential for development of interventions that can accommodate large numbers of users. They can likely increase the effectiveness of mental health professionals, enable paraprofessional and peer mental health support, and increase active self-management of problems among trauma survivors. Thus, technology-facilitated care can encompass a continuum of services ranging from professional in-person treatment that includes technologies to improve effectiveness and convenience, to technology-facilitated paraprofessional help and mutual aid among trauma survivors, to the use of technologies to support self-care of those experiencing mental health problems.

### Text messaging

Mobile mental health capabilities include the mobile phone basic function of text messaging (short messaging services or SMS). Text messaging is important given current limitations in penetration of more sophisticated devices in LMICs. Advantages specific to text messaging are many (Konrath, 2014): almost all phones have text messaging capability; text messaging is easy to learn and very widely employed; messages can be accessed at any time; and if phones are turned off, messages are delivered when they are turned back on. For researchers, it is easier and less costly to develop a text message-based study than a smartphone app study. Text messaging interventions have been found to be effective for physical activity, diabetes self-management, weight loss, smoking cessation, and medication adherence for antiretroviral therapy (Hall *et al.* 2015).

Initial studies suggest that text messaging can be used to support the management of mental health problems, but the literature is small and subject to significant methodological limitations (Berrouiguet *et al.* 2016; Watson *et al.* 2016). To date, text messaging has been used for reminders, information provision, supportive messages, and self-monitoring, and as an extension of traditional care rather than treatments in themselves (Berrouiguet *et al.* 2016). It has been used in India as a means of coaching college students as they used an app to manage anxiety (Kanuri *et al.* 2015). More work is needed to establish whether and how text messaging might offer opportunities to manage post-traumatic stress reactions and related problems during and after exposure to PTEs. Text messaging has been used to monitor PTSD symptoms among injured trauma survivors after hospital discharge (Price *et al.* 2014), suggesting that it offer a feasible way to monitor at-risk populations. Texting has also been used to encourage use of CBT skills and smartphone apps by military service members experiencing sub-threshold PTSD symptoms (Roy *et al.* 2015). Despite these early demonstrations, this modality of support has as yet remained relatively unexplored. Nonetheless, it seems likely that texted content can provide helpful education for trauma survivors and their families, and might possibly be used to increase coping skills and promote adaptive responses to trauma.

### Internet interventions

The Internet enables delivery of interactive content that can provide information, facilitate assessment, mobilize social support, strengthen self-management, and provide skills training. Research indicates that Internet interventions are effective in reducing symptoms of depression and anxiety (e.g. Saddichha *et al.* 2014) and alcohol consumption (Riper *et al.* 2007; Brief *et al.* 2013), and addressing a range of consequences of trauma (Amstadter *et al.* 2009). Web-based interventions have been developed as a response to mass traumas such as disaster (Ruggiero *et al*. 2012, 2015) and war (Bush *et al.* 2011). Internet interventions for PTSD are significantly more effective than passive controls, with medium to large effect sizes (Kuester *et al.* 2016). However, Internet interventions for trauma survivors in LMICs have received little research attention. In their review of online interventions conducted in LMICs, Arjadi *et al.* (2015) located only three randomized controlled trials. Two studies of *Ilajnafsy*, an adaptation (for use in Iraq) of Interapy, a therapist-supported, narrative writing PTSD intervention, suggest that such interventions will be helpful in LMICs (Wagner *et al.* 2012; Knaevelsrud *et al.* 2015). Knaevelsrud *et al.* (2015) showed that the online intervention reduced PTSD symptoms experienced by war-traumatized Arab residents in Iraq, suggesting that, even in unstable settings with ongoing exposure to human rights violations through war and dictatorships, technology-facilitated interventions may benefit trauma survivors. A Mandarin version of the My Trauma Recovery website, an online tool designed to improve trauma survivor coping skills (e.g. social support, self-talk, relaxation), was tested in both an urban sample exposed to a variety of traumas and with rural survivors of the 2008 Szechuan earthquake in China and PTSD symptoms improved more among those using the intervention than among controls (Wang *et al.* 2013).

### Mobile phone applications

There has been little research on phone technologies that address mental health outcomes. *PTSD Coach* is probably the most used PTSD-related app; globally, *PTSD Coach* has been downloaded over 260 000 times in 96 countries. Several studies of the app have been conducted, with some promising results (Kuhn *et al.* 2014; Owen *et al.* 2015; Miner *et al.* 2016; Possemato *et al.* 2016; Kuhn *et al.* 2017). Kuhn *et al*. (2017) studied the impact of *PTSD Coach* with 120 community trauma survivors, finding that individuals using *PTSD Coach* for three months experienced significantly greater PTSD symptom reductions than individuals in a waitlist control condition. *PTSD Coach* was not originally designed to reduce symptoms of post-traumatic stress; rather, it was conceived as a general psychoeducational resource as well as a self-help tool for reduction of acute distress. It is likely that apps that incorporate more tools to enhance skills of emotional self-management can have a significant impact on user wellbeing.

It is also possible that smartphone apps can be used to increase engagement and adherence to EBTs for PTSD and associated problems. Apps that facilitate client participation in EBTs have been developed by the National Center for PTSD. For example, *PE Coach* (Reger *et al.* 2013) and *CPT Coach* facilitate delivery of Prolonged Exposure and CPT PTSD treatments, respectively. Deployment of such apps may help enable mental health professionals in LMICs to deliver these treatments, but in addition, it might support the task-sharing movement by similarly supporting delivery of EBTs by paraprofessionals. Recent initiatives have demonstrated that paraprofessionals can successfully provide even the most complex interventions for PTSD, such as CPT (Bass *et al.* 2013).

## Cultural adaptation of interventions

If technology-facilitated interventions are to be used in settings that differ significantly from those in which they were developed and tested, it will be necessary to adapt those interventions to ‘fit’ the new users and delivery environments (Mohr *et al.* 2014). Technological interventions should be adapted, while taking into account the various dimensions of cultural adaptation identified by Bernal & Saez-Santiago (2006): language of the intervention, therapist matching, cultural symbols and sayings (metaphors), cultural knowledge or content, treatment conceptualization, treatment goals, treatment methods, and consideration of treatment context. In a review of cultural adaptations of CBT interventions for depression, Chowdary *et al.* (2014) found that adaptations predominantly reflected efforts to enhance acceptability as opposed to modifications of core content, thus maintaining fidelity to the original interventions. Their meta-analysis of 16 studies reported a large effect size of interventions adapted in this way.

It is not yet clear how or to what extent interventions developed and studied in higher-resource environments will need to be modified to ensure their effectiveness in low-resource settings and different cultural contexts. Certainly, cultural differences in perceptions of mental health and symptoms are likely to impact uptake and interaction with technologies. In a study of a text messaging intervention for depression, for example, Aguilera & Berridge (2014) observed cultural differences in perceptions of messages by Spanish and English speakers. Spanish speakers more often mentioned that receiving messages made them feel cared for and supported, while English speakers perceived messages as helping them be more aware of their mood states. The authors speculated that Spanish speakers may value collectivism and relationship connections, while English speakers may value agency and independence.

Some evidence suggests that CBT interventions for trauma survivors can work with those in very different cultural contexts, if adapted for the culture and situation of participants. Bolton *et al*. (^12,13^) tailored CBT to the needs of Burmese survivors of violence. Tailoring involved making reference to Burmese folktales, including personal anecdotes from local counselors, using local expressions to convey principles, and integrating local coping skills such as meditation, singing songs, and having tea with friends. Behavioral activation was modified to focus on activities that involved helping others, strengthening family relationships, and building connections with the community. While questions can and should be raised about the fit of interventions for specific cultures, this should be explored empirically. Bolton *et al.* (^12,13^) encountered significant concerns that the exposure techniques much used in treatment of post-traumatic stress might conflict with cultural norms by requiring their Burmese clients to reveal personal details. On the contrary, individuals receiving the intervention expressed relief after sharing their painful memories.

## Challenges and possibilities

The potential roles of technology to increase the effectiveness and reach of mental health services are now increasingly recognized, and the increased availability of Internet and mobile technologies in LMICs coupled with the potential effectiveness of low-intensity psychological interventions provides a unique opportunity to offer traumatic stress-related mental health services to a wide range of individuals in LMICs. The World Innovation Summit in Health Report of the Patel & Saxena (2014) recommended that we ‘use technology to improve access to mental health care – for example, by means of computer-assisted self-guided psychological therapies’ (p. 3). Munoz (2010) argued that, to reduce global health disparities, we need ‘non-consumable’ interventions that can be used over and over again without losing their therapeutic power. He and colleagues suggested that massive open online interventions (‘MOOIs’) can reach hundreds of thousands of individuals worldwide at no cost to users or to national healthcare systems. In a proof-of-concept study, 292 978 individuals from 168 countries visited their smoking cessation website, 18 154 enrolled in their study, and 3479 reported quitting (Muñoz *et al.* 2016).

The vision of technology-facilitated mental health response that empowers more effective self-help, strengthens the capacity of non-professionals to deliver brief low-intensity interventions, and supports delivery of evidence-based psychological interventions by mental health professionals is a compelling one that is gaining visibility. Research, especially in LMICs, is just beginning, but there is good reason to believe that effective technologies can be developed. The many obstacles to realization of this vision of care include the ongoing challenges in making technologies available to those living in low-resource environments, ensuring that privacy and information security needs are met, and funding the development and maintenance of the technologies themselves. Other key challenges include finding ways to harness social media, expanding technology-based interventions to build communities and strengthen social advocacy, and developing the systems needed for implementation of technology-facilitated mental health response.

### Getting to consumers: harnessing social media

Majorities of adult Internet users in almost all emerging and developing nations use social networking sites like Facebook and Twitter. In fact, Internet users in these countries are more likely to use social media than those in the developed world (Pew Research Center, 2016). Potentially, this engagement in social media use represents an opportunity for mobile technologies to increase reach to trauma survivors and to increase post-trauma social support.

This potential for reaching trauma survivors is well illustrated in the widespread use of social media platforms to seek information and establish the safety of loved ones during and after large disasters. For example, during the 2009 H1N1 influenza pandemic, followers of the Centers for Disease Control and Prevention Twitter ‘emergency profile’ increased in number from 65 000 to 1.2 million (Merchant *et al.* 2011). Crisis-mapping and monitoring tools like that created by the non-profit company Ushahidi have been used in response to post-election violence in Kenya, the 2010 Haiti earthquake, and in disasters occurring in Pakistan, Chile, and Japan (Peary *et al.* 2012). According to the Haiti Crisis report, these tools were quickly deployed in the aftermath of the earthquake to collect critical information and successfully distribute essential, yet limited resources (Heinzelman & Waters, 2010). Facebook's Safety Check feature is playing an increasingly vital role in disaster response by allowing users to let friends and loved ones know whether they are safe during disaster (Gleit *et al.* 2014).

Lack of social support is one of the strongest predictors of development of PTSD (Brewin *et al.* 2000) and social exclusion or ostracism after traumatic experiences is a common phenomenon (Maercker & Horn, 2013). Given that strengthening of sense of connectedness and social support is seen as an essential principle of intervention after mass trauma (Hobfoll *et al.* 2007), that social media is widely used in LMICs and following disasters, it will be helpful to explore further how social media might be used to increase support for trauma survivors and how social media interventions might be further developed.

### Technologies for community support and change

The approaches advocated above, in which individual-focused interventions are simplified for wider and more rapid delivery to those in need, may be limited in important ways. Wars, community violence, disasters, and other mass traumas impose collective hardship on groups and communities, over and above their impact on individuals. Jansen *et al.* (2015) questioned the extent to which mental health interventions should remain focused on the individual rather than addressing the suffering experienced collectively by communities. Noting that genocide in Rwanda resulted in 800 000 killed in 100 days and also destroyed Rwandan patterns of social life, they argued that interventions should emphasize social reconnection and the harnessing of collective strengths and resources. There has also been some concern that the adoption of individual mental health treatments might represent a ‘medicalization’ of distress (Clark, 2014; Whitley, 2015) and perhaps contribute to a lessened attention to the social determinants of mental health that are so important in LMICs. For example, a major cause of mental health difficulties is violence against women, including intimate partner violence, forced early marriage, honor killing, rape, and female genital mutilation (Ellsburg *et al.* 2015), and many women lack access to basic services, education, jobs, money, and power. A narrow biomedical approach to mental health is at risk of failing to address the array of factors that contribute to mental health challenges and might inadvertently devalue the effects of exposure to lifelong cumulative stress and adversity, including violence, poverty, inequality, governmental corruption, infant mortality, infectious disease, and other ills (Drake, 2015).

Given that mental health problems in LMICs are clearly related to social adversity (Tol *et al.* 2014; Whitley, 2015), it would seem advisable that technology-facilitated individual interventions should be seen as part of a more comprehensive, and more powerful, mental health response in LMICs that may be in part empowered via technology. A critical component in low-resource settings is the mobilization of mutual aid and community organization in the aftermath of traumatic events (Yates & Paquette, 2011; Patel, 2014). Potentially, technologies could be used to facilitate the building of supportive group environments to reduce both individual and social distress (Richters *et al.* 2008). They could support the formation and operation of self-help groups and mutual aid organizations. Possibly, they could be mobilized to magnify the voices of traumatized groups and strengthen advocacy to address social injustice, which can itself be seen as a mental health intervention (Patel, 2015).

Studies have shown that knowing that there are others facing similar concerns and experiencing similar mental health symptoms can be highly reassuring and can create a sense of group belonging (Harvey *et al*. 2007). Participation in online communities by individuals with mental health difficulties can help them challenge stigma and find hope and many people with mental health issues are motivated to seek mental health care after first discussing concerns with peers online (Lawlor & Kirakowski, 2014). In addition to social support, smartphones can serve a broader range of functions that can be expected to improve mental health. For example, refugees are using phones to help navigate journeys, facilitate language translation, enable access to vital services, link with friends and family, and receive ‘digital care’ provided by professionals and volunteers (Gillespie *et al.* 2016). Stempeck's (2013) description of the Boston marathon bombing illustrates the potential for a ‘digital humanitarian’ response:

When word spread of a need for housing, over 4000 offers materialized nearly instantly on a Google Forum hosted by *The Boston Globe*. The Red Cross was deluged with offers of blood donations, and tried to shift this outpouring of goodwill to future weeks. The FBI explicitly requested the crowd at the finish line share their photos and videos of the event, and a small team of startup founders then helped the authorities improve how they collected these photos to better retain valuable metadata. Around the country and world, newspapers ran the story and millions checked in with loved ones and friends….A solidarity run organized via Facebook event offered the runner community all over the planet a forum to check in and express their support for those in the locally affected community (and to intertwine the story with their own) (Stempeck, 2013, pp. 15–16).

### Implementation of technology-facilitated services

A large evaluation of the effectiveness of Internet interventions for depression in the United Kingdom National Health Service showed a limited uptake by patients and no clinical benefit in comparison with usual primary care for depression (Gilbody *et al.* 2015). In this study, standalone Internet interventions with minimal support were in fact found to be unacceptable to a large number of patients (but perceived as helpful by others; Knowles *et al.* 2015). But in a second trial (Brabyn *et al.* 2016), increasing the level of professional support offered alongside the Internet interventions was found to lead to greater user engagement and improved outcomes. The more intensive telephone support process resulted in clinical benefits over and above usual GP care. Generally, research indicates that guided Internet interventions lead to better outcomes than unguided interventions, but how support should be provided, and how often, remains unclear, and there is no clear dose-response relationship between guidance and outcome (Andersson & Titov, 2014). The importance of support to the effectiveness of technology-based interventions highlights the need to develop the systems of care in which technologies will be deployed and to establish processes of provider training and supervision for those who will support users (cf. Hill *et al.* 2014).

Research is needed to compare the effectiveness and costs of methods of training and supervising lay helpers, and to find ways of reducing training demands. Recent initiatives have demonstrated the feasibility and effectiveness of training lay workers in the face-to-face delivery of evidence-based psychological interventions. For example, Beck *et al.* (2016) proposed a dual strategy of developing both a smaller group of master trainers/supervisors and researchers and a larger group of lay workers trained in the basics of cognitive-behavioral therapy and supported by regular supervision. This method has been successfully employed in recent outcome trials conducted in LMICs (Naeem *et al.* 2011; Husain *et al.* 2014), and could be extended to training programs targeting implementation of technology-facilitated interventions.

Implementation challenges will of course go beyond issues of training and support. As highlighted by the WHO Mental Health Gap Action Programme (World Health Organization, 2010), even if local lay counselors can be trained to support delivery of interventions without loss of treatment fidelity, initial training and ongoing supervision may require significant financial and structural resources (Bockting *et al.* 2016). Innovative approaches to eHealth for LMICs will need political and financial investments in development, evaluation, and delivery of high-yield, cost-effective, and scalable interventions for these approaches to succeed. Such investments will need to address costs of Internet or mobile access for affected populations, and the development of adequately resourced organizations tasked with the development and maintenance of technological infrastructures needed for the required maintenance and hosting of online or mobile phone-based interventions (e.g. apps will need to be updated when new operating systems are released).

Interestingly, it is possible that deployment of technology-based mental health interventions in LMICs may encounter fewer obstacles compared with implementation in more developed better-resourced environments. Disadvantages of Internet-based interventions identified by Andersson & Titov (2014) included negative clinician and patient attitudes, clinician fears that their work might be threatened, and a lack of willingness of practitioners to refer patients for the interventions. These concerns seem less relevant in LMICs where there are few mental health clinicians and few services to which to refer.

## Conclusions

There is increasing recognition of the potential for technologies to be used to address global mental health problems and increase both reach and effectiveness. Research in well-resourced treatment environments has indicated the potential effectiveness of Internet and phone technologies in addressing a range of mental health problems, and there is reason to believe that they might also be effective in LMICs. In this paper, we emphasize the need, especially, to address the needs of the millions exposed to wars, ethnic conflicts, natural and human-caused disasters, and other traumatic events, which lead to a wide range of mental health problems, including post-traumatic stress reactions, depression, anxiety, and substance abuse; and given the multifaceted needs of trauma survivors, we draw attention to the need to develop trans-diagnostic and cross-problem interventions that can cut across specific disorders and target many of their needs simultaneously.

Mental health interventions that can be feasibly delivered in LMICs are being developed and implemented, but to date, Internet and phone technologies have received relatively little attention in mental health, and given the soon-to-be-expected rapid penetration of these technologies globally, it is important to actively explore their integration into mental health services. Technology should not replace the need for human interaction during the process of trauma recovery, but should be designed to increase human connection and empower both lay workers and mental health professionals to more effectively support traumatized individuals and communities. Technologies can also enhance individual self-help and provide the means through which individuals might band together in mutual aid efforts, help each other with recovery, and attain a collective voice.

Second-generation networks of phones (that include text messaging but do not provide Internet access and other advanced features of smartphones) will remain the dominant technology over the near term in many developing countries, so that simple text messaging interventions require more development and evaluation. Although it will take some years for smartphones to become widely available to low-income populations, they are the primary way in which billions of individuals in LMICs will be brought online. Potentially, phone apps can incorporate the various intervention components found in effective face-to-face (and Internet) interventions (e.g. skills training, individualized assessment, goal-setting, and self-monitoring), and there is a need to anticipate increased availability by strengthening the therapeutic efficacy of app interventions.

In all this work, evaluation will be critical. Pilot studies will need to pave the way for larger randomized controlled trials and investigations of effectiveness under real-world conditions. Interventions will need to be adapted for different cultural contexts and studies will need to be conducted across a range of countries and cultures, in different healthcare systems and societal settings (Farrington *et al.* 2014). As this work moves forward, it will be necessary to find ways of integrating quality improvement into intervention trials, to enable technology interventions to evolve more rapidly (Mohr *et al.* 2015), and more ‘agile’ research methodologies will need to be developed and applied (Patrick *et al.* 2016). The ability of technologies to facilitate measurement of outcomes should make it easier to implement quality improvement processes and conduct outcome evaluations, but it is not yet clear how it will be possible to take on the demands of developing, evaluating, and especially, providing training, supervision, and implementation support for those who want to use these technologies in their communities and countries. Technologies themselves will need to be maintained and improved across time, again requiring ongoing resources.

There are formidable technological, research, and implementation challenges associated with development and large-scale deployment in LMICs of phone and Internet mental health technologies. These challenges will require the attention of many groups, including researchers, technology developers, and those who deliver mental health services. However, work to date indicates that these technologies are indeed feasible to develop, evaluate, and deliver to those in need of services, and that they can be effective. Given the rapid uptake of mobile phones across the world, and soon-to-be-realized near universal access to more sophisticated smartphone technologies, it is important to mobilize efforts now to seize an emerging opportunity to improve the wellbeing of millions of trauma survivors exposed to stressful, abusive, and inhumane environments in LMICs.
